# Is “Culture” Needed in Aboriginal Elder Care Station? An Study from the Perspective of Service Providers

**DOI:** 10.1093/geroni/igab046.3620

**Published:** 2021-12-17

**Authors:** Li-Chuan Liu

**Affiliations:** National Taitung University, Taitung, Taitung, Taiwan (Republic of China)

## Abstract

Many studies show that cultural perspective is an important factor in caring for elderly tribal adults. To understand the level of attention that the Cultural Health Station of Indigenous People attaches to culture during its operation, this study selected Taitung County as the region of study. A qualitative focus group research method and quantitative questionnaire, we try to understand “What are the demands of elderly tribal adults?” “Do services provided by the Tribal Cultural Health Station satisfy the demands of elderly tribal adults?” and “What are the gaps between the service demands and provided to elderly tribal adults?” The results showed that service providers believe that culture is markedly important to elderly tribal adults, that culture-based care designs offered by the Tribal Cultural Health Station is currently insufficient, and that to enhance the capacity of the multiethnic Tribal Cultural Health Station, the cognition and understanding of policy makers and enforcers must be elevated.

